# A novel adenoma–carcinoma sequence- and disulfidptosis-associated gene signature for prognostic prediction and mechanistic insights in colorectal cancer

**DOI:** 10.1016/j.gendis.2026.102269

**Published:** 2026-06-03

**Authors:** Zhen Wang, Yirong Li, Runlin Gui, Yang Li, Yuanyuan Ren, Jie Liu, Wenzhen Shi, Yuyan Xiong

**Affiliations:** a Key Laboratory of Resource Biology and Biotechnology in Western China, Ministry of Education, College of Life Sciences, Northwest University, Xi'an, Shaanxi 710069, China; b Medical Research Center, Xi'an No.3 Hospital, The affiliated Hospital of Northwest University, Xi'an, Shaanxi 710018, China; c Xi'an Key Laboratory of Cardiovascular and Cerebrovascular Diseases, Xi'an No.3 Hospital, The Affiliated Hospital of Northwest University, Xi'an, Shaanxi 710018, China

Colorectal cancer (CRC) ranks among the most prevalent and lethal malignancies worldwide, with the adenoma–carcinoma sequence (ACS) being a key tumorigenic pathway.^1^ During ACS progression, colorectal epithelial cells undergo metabolic rewiring, including increased oxidative stress, glutathione demand, and actin cytoskeleton remodeling^2^^,^^3^ which may sensitize them to disulfidptosis, a newly identified regulated cell death triggered by disulfide bond accumulation and cytoskeletal collapse. Mechanistically distinct from ferroptosis and pyroptosis, disulfidptosis demonstrates potential to overcome cancer therapy resistance,^4^ yet its prognostic relevance and regulatory mechanisms in ACS-driven CRC remain largely unknown. In this study, we integrated single-cell and bulk transcriptomic datasets to identify ACS- and disulfidptosis-related genes (ACS-DRGs) associated with CRC progression and outcomes, constructed a prognostic model, characterized the immune landscape, assessed chemotherapy sensitivity, and investigated the molecular mechanisms.

Detailed materials and methods are provided in the Supplementary Materials and Methods, and the overall study workflow is illustrated in Figure S1. Transcriptomic data from TCGA and GTEx databases were harmonized using the “combat” function (Fig. S2A and S2B). Four disulfidptosis-related key genes (DRKGs)—SLC7A11, SLC3A2, RPN1, and NCKAP1,^5^ were significantly upregulated in CRC tissues (Fig. 1A; Fig. S2C). Somatic mutations occurred in 8.44% of patients, with SLC3A2 being most frequently altered (Fig. S2D). After outlier removal (Fig. 1B; Fig. S3A and S3B), differential expression analysis between 393 normal and 624 CRC tissues identified 10,732 CRC-related differentially expressed genes (CRC-DEGs) (Fig. 1C). Weighted gene co-expression network analysis (WGCNA; soft threshold = 14) generated 37 modules, 14 correlated with DRKGs (COR ≥ 0.7), yielding 6669 potential disulfidptosis-related genes (DRGs) (Fig. 1D; Fig. S3C–S3E). Single-cell RNA sequencing analysis of normal, adenoma, and carcinoma tissues identified 967 normal–adenoma DEGs (NA-DEGs), 641 adenoma–carcinoma DEGs (AC-DEGs), and 1551 normal–carcinoma DEGs (NC-DEGs) (Fig. S4A–S4E). A total of 36 ACS-DRGs were identified through intersection analysis (Fig. 1E). Univariate Cox regression identified seven prognostic ACS-DRGs (Fig. 1F). LASSO regression (Fig. 1G; Fig. S5A) followed by stepwise regression (Table S2) retained six prognostic ACS-DRGs (CXCL2, LGALS2, SELENOP, S100P, ETS2, and TIMP1). These ACS-DRGs were incorporated into a multivariable Cox regression model (Table S3), and proportional hazards assumption was confirmed (Fig. S5B). Risk scores were calculated based on coefficients to stratify CRC patients into high- and low-risk groups (Fig. S5C and S5D). In the high-risk group, CXCL2, LGALS2, SELENOP, S100P, and ETS2 were downregulated, whereas TIMP1 was upregulated (Fig. S5E). Kaplan–Meier analysis revealed significantly poorer survival in high-risk patients (Fig. 1H). Time-dependent ROC analysis yielded AUCs of 0.62, 0.67, and 0.64 for 1-, 3-, and 5-year survival, respectively (Fig. S5F). The model was validated in the TCGA and three GEO cohorts (Table S1 and Fig. S6A–S6H), demonstrating consistent survival stratification and comparable AUC values. Compared with four published CRC prognostic models, the ACS-DRGs model showed higher time-dependent AUCs and C-index values (Fig. S6I–S6L), indicating superior discriminative ability and long-term stability. Despite modest AUCs, the model independently stratified patients into distinct risk groups with significantly different outcomes (Fig. 1I), supporting its potential clinical utility for risk-adapted surveillance and treatment.

**Figure 1:**
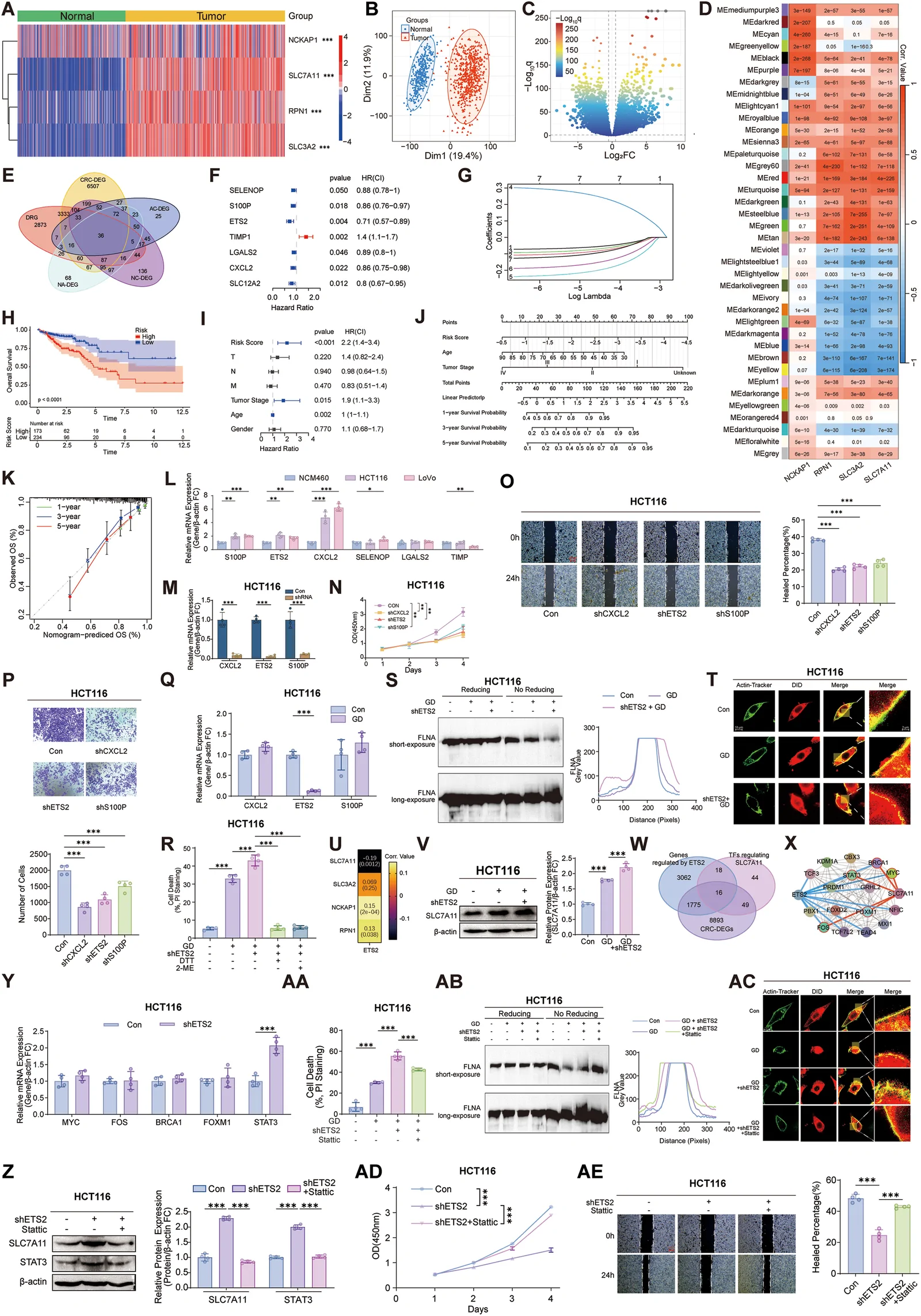
Identification of a novel adenoma–carcinoma sequence- and disulfidptosis-associated gene signature for prognostic prediction and mechanistic insights in colorectal cancer (CRC). **(A)** Heatmap analysis of differential expression of DRKGs between normal and CRC tissues. **(B)** Principal component analysis between normal and CRC groups. **(C)** Volcano plot of differentially expressed genes (DEGs) between normal and CRC samples. **(D)** Weighted Gene Co-Expression Network Analysis of DRKGs in TCGA-COADREAD cohorts. **(E)** Venn diagram showing the intersection of DRGs, CRC-DEGs, NA-DEGs, AC-DEGs, and NC-DEGs. **(F)** Forest plot of the univariate Cox regression analysis for ACS-DRGs. **(G)** LASSO Cox regression analysis of the prognostic ACS-DRGs. **(H)** Kaplan–Meier curves of the gene signature in TCGA COADREAD training cohorts. **(I)** Forest plots of the multivariate Cox regression analysis in TCGA COADREAD cohorts. **(J)** Nomogram constructed from three independent prognostic factors identified by multivariate Cox regression. **(K)** Calibration curves for 1-, 3-, and 5-year OS prediction by the nomogram. The *x*-axis represents nomogram-predicted OS; the *y*-axis represents observed OS. **(L)** mRNA expression levels of six ACS-DRGs in normal colonic epithelial cells (NCM460) and CRC cell lines (HCT116, LoVo). **(M)** qPCR validation of ACS-DRG knockdown efficiency in CRC cell lines. **(N)** CCK-8 assay measuring HCT116 cell proliferation. Statistical significance was determined using one-way ANOVA followed by Dunnett's multiple comparisons test. **(O,P)** Wound healing and transwell assays assessing migration and invasion of HCT116 cells. **(Q)** qPCR analysis of CXCL2, ETS2 and S100P mRNA levels in HCT116 cells under glucose deprivation (GD). **(R)** Cell death under GD was detected by PI staining in HCT116 cells. **(S)** Non-reducing Western blot analysis showing GD-induced shifts in electrophoretic mobility of FLNA and FLNB, indicating disulfide bond formation. **(T)** Actin-Tracker and DiD co-staining images of HCT116 cells under GD. **(U)** Pearson correlation analysis between ETS2 and DRKGs (SLC7A11, SLC3A2, NCKAP1, RPN1) in the TCGA-COADREAD cohort. **(V)** Western blot and quantification results for SLC7A11 protein levels in HCT116 cells. **(W)** Venn diagram analysis of ETS2-regulated genes and SLC7A11 regulators identified by hTFtarget and CRC-DEGs. **(X)** Protein–protein interaction (PPI) network of common interactors of common transcription factors (TFs), ETS2 and SLC7A11 using STRING. **(Y)** qPCR analysis of TF expression after ETS2 knockdown in HCT116 cells. **(Z)** Western blot analysis of SLC7A11 and STAT3 expression following STAT3 inhibition in ETS2-silenced HCT116 cells. **(AA)** Cell death assay of ETS2-silenced HCT116 cells under GD. **(AB)** Non-reducing Western blot analysis in ETS2-silenced HCT116 cells under GD with or without STAT3 inhibition. **(AC)** Actin-Tracker and DiD co-staining in ETS2-silenced HCT116 cells under GD with or without STAT3 inhibition. **(AD)** CCK-8 assays of HCT116 cells. Statistical significance was determined using one-way ANOVA followed by Dunnett's multiple comparisons test. **(AE)** Wound healing assays in HCT116 cells. Statistical significance was determined using Student's *t*-test. Error bars represent mean ± SEM. ∗*p* < 0.05, ∗∗*p* < 0.01, ∗∗∗*p* < 0.001.

Univariate (Fig. S6M) and multivariate (Fig. 1I) Cox regression analyses further identified the risk score, tumor stage, and age as independent prognostic factors. Based on these variables, a nomogram was constructed to predict overall survival in CRC patients (Fig. 1J). Calibration curves demonstrated strong concordance between nomogram-predicted and observed survival at 1-, 3-, and 5-year time points (Fig. 1K). Time-dependent C-index analysis showed that the nomogram outperformed individual predictors, achieving a C-index of 0.763 (Fig. S6N). ESTIMATE analysis revealed significantly higher stromal, immune, and overall ESTIMATE scores, along with lower tumor purity, in the high-risk group (Fig. S7A). Consistently, CIBERSORT analysis indicated reduced proportions of several immune cell subsets and an increased abundance of M0 macrophages in the high-risk group (Fig. S7B and S7C). Additionally, chemotherapy sensitivity analysis of 198 drugs using the calcPhenotype algorithm (Table S4) identified dihydrorotenone and TAF1_5496 as potentially more effective in low-risk patients, whereas AZD1332 and XAV939 were predicted to be more effective in high-risk patients (Fig. S7D).

To further examine the correlation between ACS-DRGs and CRC pathogenesis, we analyzed the expression profiles of six ACS-DRGs in the TCGA-COADREAD dataset. CXCL2, TIMP1, ETS2, and S100P were significantly upregulated in tumor tissues, whereas LGALS2 and SELENOP were downregulated (Fig. S8A). Immunohistochemical analyses further confirmed increased expression of TIMP1 and S100P and decreased expression of LGALS2 and SELENOP (Fig. S8B–S8E). Consistently, qPCR analysis demonstrated markedly elevated mRNA levels of S100P, ETS2, and CXCL2 in CRC cell lines (HCT116 and LoVo) compared with normal colonic epithelial NCM460 cells (Fig. 1L). To validate the functional involvement of ACS-DRGs in CRC progression, CXCL2, ETS2, and S100P were silenced in HCT116 and LoVo cells (Fig. 1M; Fig. S9A). Knockdown of CXCL2, ETS2, or S100P significantly suppressed cell proliferation, migration, invasion, and clonogenic capacity (Fig. 1N–P; Fig. S9B–S9F). To determine whether these genes contribute to CRC malignancy via disulfidptosis, glucose deprivation (GD) was employed to induce disulfidptosis. GD markedly reduced ETS2 but not CXCL2 or S100P expression, suggesting a specific role for ETS2 in modulating disulfidptosis (Fig. 1Q; Fig. S10A). Moreover, ETS2 knockdown exacerbated GD-induced cell death, which was rescued by the disulfidptosis inhibitors DTT and 2-ME (Fig. 1R; Fig. S10B). Disulfidptosis is characterized by excessive disulfide bond formation and actin cytoskeleton disruption.^5^ Notably, non-reducing Western blotting of FLNA and FLNB (Fig. 1S; Fig. S10C and S10D), together with fluorescence-based cytoskeletal staining (Fig. 1T; Fig. S10E), revealed increased disulfide bond accumulation and cytoskeletal disassembly in ETS2-depleted HCT116 and LoVo cells, further supporting a suppressive role of ETS2 in disulfidptosis during CRC progression.

Finally, to elucidate the underlying mechanism, correlation analysis identified SLC7A11 as inversely associated with ETS2 (Fig. 1U). GD treatment induced upregulation of SLC7A11, which was further enhanced by ETS2 knockdown, indicating that ETS2 negatively regulates SLC7A11 (Fig. 1V; Fig. S11A–S11C). Given that ETS2 functions as a transcription factor (TF), we hypothesized that this regulation occurs via intermediary TFs. Integrative analysis of ETS2-regulated genes, CRC differentially expressed genes, and SLC7A11-related TFs from the hTFtarget database identified 16 candidate TFs (Fig. 1W). Protein–protein interaction analysis highlighted BRCA1, MYC, FOXM1, FOS, and STAT3 as shared interactors of ETS2 and SLC7A11 (Fig. 1X), among which STAT3 was significantly upregulated following ETS2 knockdown (Fig. 1Y; Fig. S11D). Pharmacological inhibition of STAT3 with Stattic reversed ETS2 knockdown-induced SLC7A11 upregulation and rescued disulfidptosis, as well as the associated suppression of proliferation, migration, invasion, and clonogenic capacity (Fig. 1Z–AE; Fig. S11E–S11K, S12A–S12E), confirming that ETS2 suppresses CRC progression by inhibiting the STAT3–SLC7A11 signaling axis.

Despite providing novel insights into the interplay among the ACS, disulfidptosis, and CRC progression, several limitations should be noted. First, the findings are largely based on retrospective public datasets and *in vitro* models, warranting validation in *in vivo* systems. Second, the molecular mechanism by which ETS2 represses STAT3 activity remains to be elucidated. Third, the clinical utility of the six ACS-DRGs signature requires prospective validation in independent multicenter cohorts, with standardized risk-score thresholds and integration into existing staging systems.

In conclusion, we identified a robust ACS-DRGs prognostic signature that effectively stratifies CRC patients and predicts prognosis and immune infiltration. Experimental validation revealed that ETS2, a key ACS-DRG, promotes CRC progression by inhibiting disulfidptosis via the STAT3–SLC7A11 axis, providing mechanistic insight and potential targets for personalized therapy.

## CRediT authorship contribution statement

**Zhen Wang:** Writing – review & editing, Writing – original draft, Data curation, Conceptualization. **Yirong Li:** Validation, Data curation. **Runlin Gui:** Data curation. **Yang Li:** Validation, Software. **Yuanyuan Ren:** Validation, Data curation. **Jie Liu:** Writing – review & editing, Funding acquisition. **Wenzhen Shi:** Writing – review & editing, Supervision, Project administration, Conceptualization. **Yuyan Xiong:** Writing – review & editing, Supervision, Project administration, Funding acquisition.

## Data availability

The datasets supporting the conclusions of this article are included within the article and its additional files.

## Funding

This work was funded by the 10.13039/501100001809National Natural Science Foundation of China (No. 82570479), the Social Development Special Fund of 10.13039/501100008871Yunnan Province Science and Technology Department (China) (No. 202403AC100017), the Key Research and Development Project of the Tibet Autonomous Region (China) (No. XZ202501ZY0044), and the National Science and Technology Fund youth cultivation project of Xi'an No.3 Hospital (China) (No. Y2023qn0003).

## Conflict of interests

All authors declare no financial or non-financial competing interests.
