# Supplementary material for: A novel adenoma–carcinoma sequence- and disulfidptosis-associated gene signature for prognostic prediction and mechanistic insights in colorectal cancer
Source: Genes Dis. 2026 Jun 3;14(2):102269. doi: 10.1016/j.gendis.2026.102269 (PMC13634562; doi:10.1016/j.gendis.2026.102269)
Supplement: Supplementary file 1 [file mmc1.docx]

**Figure S1. The flowchart of the overall analytical workflow and key methodologies employed.**

**Figure S2. Genetic characteristics of DRKGs in TCGA COADREAD cohorts.** (A) Significant batch effects were observed in the TCGA cohort and GTEx cohorts. (B) The batch effect was removed. (C) Violin Diagram of the DRKGs expression profiles in TCGA COADREAD cohorts. (D) Somatic mutations of DRKGs in 616 TCGA COADREAD cohorts; Each column in the waterfall plot represents the mutation type for each patient, the tumor mutation burden (TMB) for each patient is shown in the top half, the mutation frequency and mutation type ratio of DRKGs are shown on the right, and the proportion of different base transitions is shown below. ***p<0.001.

**Figure S3. Weighted Gene Co-Expression Network Analysis to identify DRGs.** (A) 31 samples were unveiled aberrant gene expression. (B) After excluding these samples, 393 normal and 624 tumor samples were retained. (C-E) Weighted Gene Co-Expression Network Analysis of DRKGs in TCGA-COAADREAD. 14 modules were most associated with DRKGs, of which 6669 genes were extracted.

**Figure S4. Annotation of cell subsets and identification of differentially expressed genes**. (A) The elbow map helps decide the number of PCs for PCA dimension reduction. (B) Cells were annotated into 13 different types of cells. (C-E) Analysis of DEGs between (C) Normal and Adenoma, (D) Adenoma and Carcinoma, and (E) Normal and Carcinoma samples.

**Figure S5. Construction of the ACS-DRGs-related prognostic model in the TCGA training cohort.** (A) Cross-validation for tuning parameter (lambda) screening in the LASSO regression analysis. (B) The proportional hazards assumption test. (C, D) The distributions of the risk score, survival time, and status of patients in TCGA COADREAD training cohorts. (E) Heatmap of the gene-expression profiles of the ACS-DRGs signatures in TCGA-COADREAD training cohorts. (F) The time-dependent ROC curves of the prognostic gene signature in TCGA COADREAD training cohorts. ***p<0.001.

**Figure S6. Validation of risk score model.** (A, C, E, G) Survival analysis between the high- and low-risk score groups in the validation cohorts. (A) TCGA COADREAD validating cohorts; (C) GSE39582; (E) GSE41258; (G)GSE17538. Log-rank test. (B, D, F, H) Time-dependent ROC curve analysis of the risk score in the validation cohorts. (B) TCGA COADREAD validating cohorts; (D) GSE39582; (F) GSE41258; (H) GSE17538. (I–K) Time-dependent ROC curve analyses comparing the predictive performance of the ACSDRGs model with four representative published prognostic models in using the TCGA-COADREAD cohort at 1, 3, and 5 years: Model A, Model B, Model C, and Model D. (L) Time-dependent concordance index (C-index) curves comparing the dynamic predictive accuracy among models across the entire survival period. (M) Forest plots of the univariate Cox regression analysis in TCGA COADREAD cohorts. (N) C-index curve analysis of nomogram and other prognostic factors.

**Figure S7.** **Analysis of immune status and drug sensitivity for TCGA COADREAD patients combined with ACS-DRGs signature.** (A) Comparison of the stromal score, immune score, ESTIMATE score, and tumor purity between the high-risk group and the low-risk group in TCGA COADREAD cohorts. (B, C) The distribution of the 22 tumor-infiltrating immune cells in the TCGA COADREAD cohort. (D) Imputed sensitivity of four drugs in TCGA COADREAD cohorts. **p<0.01, and ***p<0.001.

**Figure S8. Expression profiles of ACS-DRGs in CRC cohorts.** (A) Differential mRNA expression levels of ACS-DRGs between tumor and normal samples. (B–E) Representative immunohistochemistry (IHC) images of (B) LGALS2, (C) TIMP1, (D) S100P, and (E) SELENOP proteins in normal and tumor tissues, obtained from the Human Protein Atlas (HPA). Protein expression levels were categorized based on staining intensity and the percentage of positively stained cells: negative (no staining), weak (<25% positive cells), moderate (25–75% positive cells), and strong (>75% positive cells). ***p<0.001.

**Figure S9. Knockdown of CXCL2, ETS2 and S100P suppresses cell migration, invasion and proliferation in CRC cell lines.** HCT116 and LoVo cells were transduced with pLKO.1-scramble (control, Con), pLKO.1-CXCL2 shRNA (shCXCL2), pLKO.1-ETS2 shRNA (shETS2), or pLKO.1-S100P shRNA (shS100P) to silence target genes. (A) qPCR validation of ACS-DRG knockdown efficiency in CRC cell lines. (B) Cell proliferation assessed by CCK-8 assay in LoVo cells. Statistical significance was determined using one-way ANOVA followed by Dunnett’s multiple comparisons test. (C) Wound healing assays in LoVo cells. (D) Transwell invasion assays in LoVo cells. (E, F) Colony formation assays in HCT116 (E) and LoVo (F) cells. Statistical analysis was performed using Student’s t-test (*p < 0.05, **p < 0.01, ***p < 0.001). Error bars represent mean ± SEM.

**Figure S10. ETS2 knockdown enhances disulfidptosis susceptibility in CRC cell lines.** HCT116 and LoVo cells were transduced with pLKO.1-shRNA (shETS2, shCXCL2 or shS100P) or transduced with pLKO.1-TRC (control, Con), and exposed to glucose deprivation (GD) to induce disulfidptosis. (A) qPCR analysis of CXCL2, ETS2 and S100P mRNA levels in LoVo cells under GD. (B) Cell death under GD was detected by PI staining in LoVo cells. (C-D) Non-reducing Western blot analysis detecting a GD-induced shift in the electrophoretic mobility of actin cytoskeleton-associated proteins FLNA and FLNB in LoVo & HCT116 cells, indicative of disulfide bond formation. (E) Actin-Tracker and DiD co-staining images of LoVo cells under GD. Statistical analysis was performed using Student’s t-test. Error bars represent mean ± SEM. ***p < 0.001.

**Figure S11. ETS2 negatively regulates disulfidptosis through the STAT3–SLC7A11 axis in CRC cells.** (A-B) qPCR analysis of SLC7A11 expression in HCT116 and LoVo cells under glucose deprivation (GD) with or without ETS2 knockdown. (C) Western blot and quantification results for SLC7A11 protein levels in LoVo cells. (D) qPCR analysis of TFs expression after ETS2 knockdown in LoVo cells. (E-G) qPCR and Western blot analysis of SLC7A11 and STAT3 expression following STAT3 inhibition in ETS2-silenced HCT116 (E) and LoVo (F-G) cells. (H) Cell death assay of ETS2-silenced and LoVo cells under GD with or without STAT3 inhibition. (I-J) Non-reducing Western blot analysis of FLNA and FLNB in ETS2-silenced LoVo&HCT116 cells under GD with or without STAT3 inhibition. (K) Actin-Tracker and DiD co-staining in ETS2-silenced LoVo cells under GD with or without STAT3 inhibition. Statistical significance was determined using Student’s t-test. Error bars represent mean ± SEM. **p < 0.01, ***p < 0.001.

**Figure S12. Inhibition of STAT3 rescues ETS2 knockdown-induced decreases in CRC proliferation, migration, and invasion.** (A) CCK-8 assays of LoVo cells. Statistical significance was determined using one-way ANOVA followed by Dunnett’s multiple comparisons test. (B) Wound healing assays in LoVo cells. (C, D) Transwell invasion assays in HCT116 (C) and LoVo (D) cells. (E) Colony formation assays of CRC cells. Statistical significance was determined using Student’s t-test. Error bars represent mean ± SEM. ***p < 0.001.
