# Supplementary material for: A novel adenoma–carcinoma sequence- and disulfidptosis-associated gene signature for prognostic prediction and mechanistic insights in colorectal cancer
Source: Genes Dis. 2026 Jun 3;14(2):102269. doi: 10.1016/j.gendis.2026.102269 (PMC13634562; doi:10.1016/j.gendis.2026.102269)
Supplement: Supplementary file 2 [file mmc2.docx]

**Materials and methods**

**Acquisition and processing of transcriptomic data**

Transcriptome data analyzed in this study were obtained from the Colon Adenocarcinoma and Rectum Adenocarcinoma (COADREAD) dataset of the TCGA database (<https://portal.gdc.cancer.gov/>) and the GTEx-Sigmoid dataset from The Genotype-Tissue Expression (GTEx) database (<https://www.gtexportal.org/>). Gene expression count matrices and Transcripts Per Million (TPM) matrices for all samples were downloaded from both databases. Count matrices were utilized for all differential expression analyses, while log2-transformed TPM matrices were used for other gene expression-related analyses. The ‘combat’ function in the “sva” package in R was employed to adjust for batch effects between TCGA and GTEx cohort^1;2^. Principal component analysis (PCA) was performed both pre- and post-correction to visualize and determine batch effects^3^. To validate the accuracy of our prognostic prediction model, three validation datasets (GSE39582^4^, GSE41258^5;6^ and GSE17538^7-10^) were obtained from the Gene Expression Omnibus (GEO) database (<https://www.ncbi.nlm.nih.gov/geo/>)^11^. Clinical features for TCGA and GEO patients was retrieved from their respective databases.

**Analysis of single-cell transcriptomic data**

We obtained the single-cell RNA sequencing (scRNA-seq) dataset GSE161277 from the GEO database, comprising a total of 13 samples, including blood, normal, adenoma, and carcinoma samples from four CRC patients^12^. Subsequently, we focused our analysis on normal, adenoma, and carcinoma samples from three patients for further exploration. Quality Control (QC) of scRNA-seq data was conducted using the “Seurat” R package^13^. We retained cells with less than 25% mitochondrial genes, detecting between 200 and 7000 genes per cell, and each gene expressed in at least three cells to ensure high-quality scRNA-seq data. Post-QC, a total of 12,417 normal cells, 9,191 adenoma cells, and 10,227 carcinoma cells remained, resulting in 31,835 qualified cells for subsequent analysis. To integrate scRNA-seq data from different patients, we performed logarithmic normalization of gene expression matrix for each cell. Following normalization, the ‘FindVariableFeatures’ function provided by the “Seurat” package, using the default vst method, was employed to select the top 4000 genes with the highest variance as highly variable genes. To address batch effects between different datasets originating from different patients, we utilized the ‘FindIntegrationAnchors’ function in the Seurat package to identify anchor genes that bridged different datasets. Subsequently, the ‘IntegrateData’ function was used to integrate the datasets based on the identified anchor genes, generating an integrated assay matrix. After data integration, we computed cell cycle scores to ensure consistency across samples. The data were then standardized and scaled, with scaling parameters ‘vars.to.regress’ selecting percent.mt and nFeature. When determining the number of principal components (PCs) for clustering, the elbow method was employed for decision-making. Finally, PCA, clustering, and dimensionality reduction analyses were performed on the scaled scRNA-seq data to explore the structure and heterogeneity of the dataset. Cell annotation was conducted based on the cell markers used in the original study of GSE161277, as detailed in the original research.

**Weighted Gene Co-expression Network Analysis**

Weighted Gene Co-expression Network Analysis (WGCNA) is a bioinformatics method used to analyze gene expression data with the aim of identifying co-expression patterns between genes and key gene modules in biological processes and regulatory networks^14^. Key genes related to disulfidptosis (DRKGs) were sourced from a previous study^15^. Remarkably, SLC7A11, solute carrier family 3 member 2 (SLC3A2), recombinant ribophorin 1 (RPN1), and NCK-associated protein 1 (NCKAP1) were identified as the four pivotal genes in regulating disulfidptosis. In this study, we implemented WGCNA using the “WGCNA” package in R and identified gene modules associated with DRKGs expression^16^. Subsequently, we examined the genes within these modules to determine their function.

**Identification of differentially expressed genes in Adenoma-Carcinoma Sequence**

The ‘FindMarkers’ function in the “Seurat” package was employed to identify differentially expressed genes (DEGs) between different cell populations in normal, adenoma, and carcinoma samples^13^. Given the well-established normal-adenoma-carcinoma sequence as a model for the progression of sporadic CRC, where adenomas serve as the primary precursor lesions leading to carcinoma, we utilized the ‘FindMarkers’ function to determine DEGs for each specific cell type within these categories^17^. In this study, we defined these DEGs as normal-adenoma DEGs (NA-DEGs), adenoma-carcinoma DEGs (AC-DEGs), and normal-carcinoma DEGs (NC-DEGs). These identified genes are likely to play critical roles in the development of CRC, contributing valuable insights into the molecular mechanisms underlying the normal-adenoma-carcinoma sequence.

**Differential expression analysis**

We performed gene differential expression analysis between tumor and normal groups in the transcriptome data using the “DESeq2” package in R, visualizing the results through a volcano plot. We specifically utilized an unnormalized count expression matrix, a choice made to enable DESeq2 to effectively manage any discrepancies across samples arising from variations in read counts^18^. This analysis identified genes with an adj.p less than 0.05 and a fold change (FC) greater than 1.5 as statistically significant DEGs. The intersection of DEGs, disulfidptosis-related genes (DRGs), and the adenoma-carcinoma sequence-related genes, as depicted in the Venn diagram, was selected for further analysis.

**Establishment of prognostic risk score model**

We utilized univariate Cox regression analysis to identify ACS-DRGs related to prognosis in CRC patients. To prevent overfitting, we employed Lasso Cox regression analysis for dimensionality reduction and stepwise regression for further eliminating redundant model signatures. The minimum penalty parameter (λ) of the Lasso Cox model was determined through ten-fold cross-validation to select variables. The implementation of these regression analyses relies on R packages, including “glmnet” for Lasso Cox regression and “survival” for univariate and multivariate Cox regression^19;20^. In this study, we used the TCGA COADREAD cohort, consisting of 625 patients, for analysis. Samples with OS time of less than 30 days or missing survival information were excluded, resulting in 582 remaining cases. These cases were randomly divided into training and validating cohorts at a ratio of 7:3. A multivariable Cox regression analysis was conducted using the samples from the training set to establish a prognostic risk score model. Additionally, three CRC transcriptomic datasets from the GEO database served as external validation datasets for the predictive model. The risk score of each sample was calculated using the formula:

$$RiskScore=\sum expr_{i}*coef_{i}$$

where “coef” represents the regression coefficients of the ACS-DRGs signature, and “expr” denotes the expression values of these genes. Based on the cutoff value of the risk score, all samples were classified into low- and high-risk groups. Kaplan-Meier analysis employing the log-rank test was conducted to compare the OS differences between the low- and high-risk groups^21^. Furthermore, time-dependent Receiver Operating Characteristic (ROC) curves were plotted to assess the prognostic accuracy of the risk score model. Finally, both univariate and multivariate Cox analyses were performed to determine the independent predictive capability of the model. To comprehensively assess the predictive performance and robustness of the ACSDRGs-based prognostic model, we conducted head-to-head comparisons with four previously published colorectal cancer (CRC) prognostic signatures, including Model A^22^, Model B^23^, Model C^24^, and Model D^25^. For each model, risk scores were computed in the TCGA-COADREAD cohort using the gene coefficients and formulas provided in the respective publications.

**Multivariable Cox regression analysis**

Clinical variables available in the TCGA-COADREAD cohort, including age, sex, tumor stage, T/N/M classification, surgical approach, adjuvant therapy, and bowel obstruction, were initially screened for inclusion in the multivariable Cox regression. Variables with missing data exceeding 50% were excluded to minimize bias. Age and overall tumor stage were retained because they are well-established independent prognostic factors for CRC and showed minimal missingness (<5%) and significant associations with overall survival in univariate analyses (p < 0.05). These variables, together with the calculated risk score, were entered into the final multivariable Cox model.

**Nomogram construction and evaluation**

To accurately forecast OS in CRC patients, we integrated clinical attributes into the risk score model, resulting in a nomogram with three pivotal features: patients’ age, tumor stage, and prognostic risk score^26^. During the validation phase, we used the Concordance Index (C-Index) as a robust metric to assess predictive accuracy. The C-Index measures the concordance of the model, ranging from 0.5 to 1.0. A value of 0.5 indicates discordance with actual outcomes, while 1.0 signifies perfect concordance, demonstrating excellent predictive performance. Calculating the C-Index scores allowed us to quantitatively assess predictive capabilities of the model. Additionally, we utilized calibration curves to evaluate predictive accuracy, comparing predicted probabilities to observed survival rates. These curves illustrate the correlation between the prognostic probabilities generated by the model and the survival rates observed empirically, offering valuable insights into the precision and reliability of predicting patient prognoses.

**Tumor immune microenvironment landscape**

We employed the “ESTIMATE” package to ascertain the relative abundances of stromal cells, immune cells, and tumor cells within TCGA COADREAD samples. ESTIMATE provides us with scores for tumor purity, the level of stromal cells, and the infiltration level of immune cells in tumor tissues based on gene expression data^27^. CIBERSORT leverages gene expression data to estimate the relative abundances of distinct immune cell subtypes within complex tissue samples^28^. These subtypes encompass a comprehensive range, including naive B cells, memory B cells, plasma cells, CD8^+^ T cells, naive CD4^+^ T cells, resting CD4^+^ memory T cells, and activated CD4^+^ memory T cells, totaling 22 distinct immune cell types. Within the TCGA COADREAD cohort, we employed the CIBERSORT algorithm to determine the infiltration proportions of these 22 immune cell types. Subsequently, we compared these scores and proportions between low- and high-risk groups to elucidate variations in immune cell infiltration under different ACS-DRGs regulatory patterns.

**Drug sensitivity profiling and analysis**

In our pursuit to determine the sensitivity of the samples to chemotherapy agents, we conducted drug sensitivity analysis using the ‘calcPhenotype’ function from the “OncoPredict” package^29^. To train the model, we employed data sourced from the Genomics of Drug Sensitivity in Cancer (GDSC) database, specifically utilizing the GDSC2 dataset^30^. The GDSC2 dataset is a comprehensive repository of pharmacological information, containing half-maximal inhibitory concentration (IC50) values for 198 distinct compounds across 809 cell lines, along with gene expression data. The ‘calcPhenotype’ algorithm provided imputed sensitivity scores for each sample concerning the 198 compounds investigated. Lower scores indicate higher sensitivity of the sample to the compounds.

**Cell culture**

The human CRC cell lines (LoVo, HCT116) utilized in the study were procured from ProCell (Wuhan, China). Unless explicitly stated otherwise, the default culture medium employed was DMEM (Sigma, USA) supplemented with an additional 10% fetal bovine serum (FBS) (Gemini, USA) and 1% Penicillin-Streptomycin solution (100X, Beyotime). Cells were maintained in a humidified environment at 37 °C with 5% CO2. Furthermore, in our experimental setup, we employed short hairpin RNA (shRNA) technology for targeted gene knockdown, which was facilitated by lentiviral DNA transfection. This method was chosen for its efficiency and specificity in silencing the expression of particular genes, thereby enhancing the precision of our investigative approach.

**Generation of lentiviral particles**

To generate lentiviral particles, HEK293T cells were co-transfected with the empty vector plasmid pLKO.1 containing the shRNA sequence, packaging plasmid (psPAX2) and envelope plasmid (pMD2.G). Post-transfection, lentiviral particles were collected from the supernatant of HEK293T cell cultures at 24h, 48h, and 72h intervals. HCT116 and LoVo cells were transduced with lentivirus for 24 hours once cell proliferation reached 80%, with 250 μL of lentivirus added to each 6-well plates. The primer sequences used were:

The targeting sequences for hGIGYF2-shRNA, hSTAU1-shRNA and hLAMTOR4-shRNA are indicated in boldface below (only the sense strand is shown):

pLKO.1-hCXCL2-F: 5′-CCGG**GCATCGCCCATGGTTAAGAAA**CTCGAGTTTCTTAACCATGGGCGATGCTTTTTG-3′

pLKO.1-hS100P-F: 5′-CCGG**GACAAGGATGCCGTGGATAAA**CTCGAGTTTATCCACGGCATCCTTGTCTTTTTG-3′

pLKO.1-hETS2-F: 5′-CCGG**CCTGACTTTGTGGGTGACATT**CTCGAGAATGTCACCCACAAAGTCAGGTTTTTG-3′

**Cell proliferation assays**

Cell proliferation assays were performed using the Cell Counting Kit-8 (Seven Biotech, Beijing, China). In 96-well plates, each well was seeded with 100 μL of culture medium containing approximately 2000 cells for the proliferation experiments. Cell proliferation was assessed at 24, 48, 72, and 96 hours of incubation. For the measurements, 10 μL of CCK-8 solution was added to each well. After co-culturing with cells for 1 hour, the absorbance at a wavelength of 450 nm was determined using a microplate reader (Bio-Rad Laboratories, Hercules, CA, USA) to quantify the level of cell proliferation.

**Wound healing assay**

The migratory ability of CRC cells was evaluated in vitro through wound healing assay. Cells were seeded overnight in a 6-well plate, and when reached 90% confluence, a gentle scratch was created using a sterile 200 μL pipette tip. Subsequently, the medium was replaced with low-serum medium (DMEM containing 0.2% FBS) and further incubated at 37 °C for 24 hours. Cell wound images were observed and captured at 0 and 24 hours using an optical microscope (Olympus Corp., Tokyo, Japan), followed by quantitative analysis of the scratch area using Image J2.

**Colony formation assay**

Approximately 500 cells were seeded in each well of 6-well plates and cultured for approximately two weeks. Colony formation was observed under a microscope. When the cell counts within a colony reached approximately 50, the colony was fixed in paraformaldehyde (4%) for 30 minutes, followed by staining with 0.2% crystal violet (Sigma, St. Louis, MO, USA). The colonies exhibited a bluish-purple color due to the influence of crystal violet, and colony quantification was performed using ImageJ2 software.

**Transwell invasion assay**

For the assessment of CRC cell invasion capability, approximately 50,000 cells in 200 μL of serum-free culture medium were seeded in the upper chamber, while 600 μL of culture medium supplemented with 20% FBS was placed in the lower chamber. After 24 hours of incubation, non-invading cells in the upper chamber were removed using a sterile cotton swab, and invading cells on the underside of the invasion membrane were fixed with paraformaldehyde (4%) and stained with crystal violet (0.25%), followed by drying and imaging.

**Western Blotting**

Cells were lysed in RIPA buffer supplemented with protease inhibitors, and total protein concentration was determined using the Pierce BCA Protein Assay Kit (Seven, Beijing, China). For electrophoresis, 20 μg of denatured protein lysates were subjected to SDS-PAGE on 8–12% polyacrylamide gels. Half of the samples were mixed with standard reducing loading buffer containing DTT, while the remaining samples were prepared with non-reducing loading buffer lacking DTT, to enable non-reducing electrophoresis. Proteins were then transferred onto nitrocellulose membranes (Immobilon-P; Millipore, Bedford, MA, USA).

Membranes were blocked with 5% non-fat milk in TBST for 1 h at room temperature and subsequently incubated with primary antibodies either for 2 h at room temperature or overnight at 4 °C. After thorough washing, membranes were incubated with HRP-conjugated secondary antibodies for 1.5 h at room temperature. Immunoreactive bands were visualized using an enhanced chemiluminescence (ECL) detection kit (ABclonal, Wuhan, China) and captured with the Tanon 5200 Luminescent Imaging System. Band intensities were quantified using ImageJ software (NIH, USA).

**Real-time PCR (RT-PCR)**

Total RNA was extracted using Trizol reagent (Thermo Fisher Scientific, Waltham, MA, USA) following the manufacturer’s protocol. Subsequently, 1 μg of total RNA from each cell line was reverse-transcribed into cDNA using the ABScript Neo RT Master Mix for qPCR with gDNA Remover (ABclonal, Wuhan, China). Quantitative real-time PCR (qRT-PCR) was performed on a Bio-Rad CFX96 Real-Time PCR Detection System (Bio-Rad, USA) with Universal SYBR Green Fast qPCR Mix (ABclonal, Wuhan, China) under the following cycling conditions: 94 °C for 30 s, followed by 45 cycles. Relative gene expression levels were calculated using the 2^–ΔΔCt^ method, with β-actin (ACTB) serving as the internal control.

The primer sequences used for qRT-PCR are listed below (5′–3′; h: human):

| **Target Gene** | **Forward** | **Reverse** |
| --- | --- | --- |
| hS100P | CTCAAGGTGCTGATGGAGAAGG | GAACTCACTGAAGTCCACCTGG |
| hCXCL2 | GGCAGAAAGCTTGTCTCAACCC | CTCCTTCAGGAACAGCCACCAA |
| hETS2 | ACTCCGCCAACTGTGAATTGCC | CCACTGGCATACCTGTTGCTCA |
| hTIMP1 | GGAGAGTGTCTGCGGATACTTC | ACAGTAGCCAAAGATACACGTTTAC |
| hLGALS2 | GGGCAAGAACAACGGGAAGATC | CCTGTTGGGAAAAGTCAGCTCG |
| hSEPP1 | CATCTTGGTTTGCCTTTTTCCTTC | GCAGGTAGTGATGTGCAAGAGTC |
| hSLC7A11 | TCCTGCTTTGGCTCCATGAACG | AGAGGAGTGTGCTTGCGGACAT |
| hSTAT3 | CTTTGAGACCGAGGTGTATCACC | GGTCAGCATGTTGTACCACAGG |
| hMYC | CCTGGTGCTCCATGAGGAGAC | CAGACTCTGACCTTTTGCCAGG |
| hFOS | GCCTCTCTTACTACCACTCACC | AGATGGCAGTGACCGTGGGAAT |
| hBRCA1 | CTGAAGACTGCTCAGGGCTATC | AGGGTAGCTGTTAGAAGGCTGG |
| hFOXM1 | TCTGCCAATGGCAAGGTCTCCT | CTGGATTCGGTCGTTTCTGCTG |

**Cell death assays**

To assess cell death, cells were seeded in 6-well plates one day prior to treatment. Following incubation in specialized media with or without the indicated compounds, cells were harvested by trypsinization and collected into 1.5 mL microcentrifuge tubes. After a single wash with phosphate-buffered saline (PBS), cells were resuspended in cold PBS containing 1 μg/mL propidium iodide (PI, Beyotime, Shanghai, China). Dead cells (PI-positive) were subsequently analyzed by flow cytometry using fluorescence-activated cell sorting (FACS).

**Fluorescent staining of actin filaments and cellular membrane.**

Following treatment, cells on chamber slides were washed once with PBS and fixed at room temperature with 4% paraformaldehyde in 1× PBS for 10 minutes. Fixed cells were then incubated for 30 minutes at room temperature, protected from light, with Deep Red Phalloidin (Beyotime, Shanghai, China) and the plasma membrane dye DiD (Beyotime, Shanghai, China), without permeabilization. All fluorescence images were acquired using a confocal laser scanning microscope (Leica Microsystems CMS GmbH, Germany).

**Statistical analysis**

Statistical analysis and visualization of results were performed using R software (version 4.3.1). All experimental data are presented as mean ± standard deviation (SD) of three independent replicates. Statistical significance between two sets of data was assessed using the Student’s t-test, while comparisons among more than two groups were evaluated through analysis of variance (ANOVA) followed by Dunnett’s multiple comparison test. A p-value<0.05 was considered statistically significant. Asterisks denote significant differences between groups (*p<0.05; **p<0.01; ***p<0.001).

**References**

1.	Leek JT, Johnson WE, Parker HS, Jaffe AE, Storey JD. The sva package for removing batch effects and other unwanted variation in high-throughput experiments. *Bioinformatics.* 2012;28(6):882-883. doi.org/10.1093/bioinformatics/bts034

2.	Leek JT JW, Parker HS, Fertig EJ, Jaffe AE, Zhang Y, Storey JD, Torres LC. sva: Surrogate Variable Analysis. <https://bioconductor.org/packages/sva>. Published 2023. Accessed.

3.	Lever J, Krzywinski M, Altman N. Principal component analysis. *Nature Methods.* 2017;14(7):641-642. doi.org/10.1038/nmeth.4346

4.	Marisa L, de Reynies A, Duval A, et al. Gene expression classification of colon cancer into molecular subtypes: characterization, validation, and prognostic value. *PLoS Med.* 2013;10(5):e1001453. doi.org/10.1371/journal.pmed.1001453

5.	Sheffer M, Bacolod MD, Zuk O, et al. Association of survival and disease progression with chromosomal instability: a genomic exploration of colorectal cancer. *Proc Natl Acad Sci U S A.* 2009;106(17):7131-7136. doi.org/10.1073/pnas.0902232106

6.	Martin ML, Zeng Z, Adileh M, et al. Logarithmic expansion of LGR5(+) cells in human colorectal cancer. *Cell Signal.* 2018;42:97-105. doi.org/10.1016/j.cellsig.2017.09.018

7.	Williams CS, Bernard JK, Demory Beckler M, et al. ERBB4 is over-expressed in human colon cancer and enhances cellular transformation. *Carcinogenesis.* 2015;36(7):710-718. doi.org/10.1093/carcin/bgv049

8.	Smith JJ, Deane NG, Wu F, et al. Experimentally derived metastasis gene expression profile predicts recurrence and death in patients with colon cancer. *Gastroenterology.* 2010;138(3):958-968. doi.org/10.1053/j.gastro.2009.11.005

9.	Chen MS, Lo YH, Chen X, et al. Growth Factor-Independent 1 Is a Tumor Suppressor Gene in Colorectal Cancer. *Mol Cancer Res.* 2019;17(3):697-708. doi.org/10.1158/1541-7786.MCR-18-0666

10.	Freeman TJ, Smith JJ, Chen X, et al. Smad4-mediated signaling inhibits intestinal neoplasia by inhibiting expression of beta-catenin. *Gastroenterology.* 2012;142(3):562-571 e562. doi.org/10.1053/j.gastro.2011.11.026

11.	Edgar R, Domrachev M, Lash AE. Gene Expression Omnibus: NCBI gene expression and hybridization array data repository. *Nucleic Acids Res.* 2002;30(1):207-210. doi.org/10.1093/nar/30.1.207

12.	Zheng X, Song J, Yu C, et al. Single-cell transcriptomic profiling unravels the adenoma-initiation role of protein tyrosine kinases during colorectal tumorigenesis. *Signal Transduct Target Ther.* 2022;7(1):60. doi.org/10.1038/s41392-022-00881-8

13.	Hao Y, Hao S, Andersen-Nissen E, et al. Integrated analysis of multimodal single-cell data. *Cell.* 2021;184(13):3573-3587 e3529. doi.org/10.1016/j.cell.2021.04.048

14.	Zhang B, Horvath S. A general framework for weighted gene co-expression network analysis. *Stat Appl Genet Mol Biol.* 2005;4:Article17. doi.org/10.2202/1544-6115.1128

15.	Liu X, Nie L, Zhang Y, et al. Actin cytoskeleton vulnerability to disulfide stress mediates disulfidptosis. *Nat Cell Biol.* 2023;25(3):404-414. doi.org/10.1038/s41556-023-01091-2

16.	Langfelder P, Horvath S. WGCNA: an R package for weighted correlation network analysis. *BMC Bioinformatics.* 2008;9:559. doi.org/10.1186/1471-2105-9-559

17.	Lynch HT, De La Chapelle A. Hereditary Colorectal Cancer. *New England Journal of Medicine.* 2003;348(10):919-932. doi.org/10.1056/nejmra012242

18.	Love MI, Huber W, Anders S. Moderated estimation of fold change and dispersion for RNA-seq data with DESeq2. *Genome Biol.* 2014;15(12):550. doi.org/10.1186/s13059-014-0550-8

19.	Friedman J, Hastie T, Tibshirani R. Regularization Paths for Generalized Linear Models via Coordinate Descent. *J Stat Softw.* 2010;33(1):1-22. <https://www.ncbi.nlm.nih.gov/pubmed/20808728>.

20.	Tay JK, Narasimhan B, Hastie T. Elastic Net Regularization Paths for All Generalized Linear Models. *J Stat Softw.* 2023;106. doi.org/10.18637/jss.v106.i01

21.	Goel MK, Khanna P, Kishore J. Understanding survival analysis: Kaplan-Meier estimate. *Int J Ayurveda Res.* 2010;1(4):274-278. doi.org/10.4103/0974-7788.76794

22.	Hu D, Zhou Z, Wang J, Zhu K. Screening of ferroptosis-related genes with prognostic effect in colorectal cancer by bioinformatic analysis. *Front Mol Biosci.* 2022;9:979854. doi.org/10.3389/fmolb.2022.979854

23.	Liu R, Wang Y, Bu J, et al. Construction and Validation of Novel Ferroptosis-related Risk Score Signature and Prognostic Prediction Nomogram for Patients with Colorectal Cancer. *Int J Med Sci.* 2024;21(6):1103-1116. doi.org/10.7150/ijms.91446

24.	Wang X, Xu Y, Dai L, et al. A novel oxidative stress- and ferroptosis-related gene prognostic signature for distinguishing cold and hot tumors in colorectal cancer. *Front Immunol.* 2022;13:1043738. doi.org/10.3389/fimmu.2022.1043738

25.	Yue T, Chen S, Zhu J, et al. The aging-related risk signature in colorectal cancer. *Aging (Albany NY).* 2021;13(5):7330-7349. doi.org/10.18632/aging.202589

26.	Jalali A, Alvarez-Iglesias A, Roshan D, Newell J. Visualising statistical models using dynamic nomograms. *PLOS ONE.* 2019;14(11):e0225253. doi.org/10.1371/journal.pone.0225253

27.	Yoshihara K, Shahmoradgoli M, Martinez E, et al. Inferring tumour purity and stromal and immune cell admixture from expression data. *Nat Commun.* 2013;4:2612. doi.org/10.1038/ncomms3612

28.	Chen B, Khodadoust MS, Liu CL, Newman AM, Alizadeh AA. Profiling Tumor Infiltrating Immune Cells with CIBERSORT. *Methods Mol Biol.* 2018;1711:243-259. doi.org/10.1007/978-1-4939-7493-1_12

29.	Maeser D, Gruener RF, Huang RS. oncoPredict: an R package for predicting in vivo or cancer patient drug response and biomarkers from cell line screening data. *Brief Bioinform.* 2021;22(6). doi.org/10.1093/bib/bbab260

30.	Yang W, Soares J, Greninger P, et al. Genomics of Drug Sensitivity in Cancer (GDSC): a resource for therapeutic biomarker discovery in cancer cells. *Nucleic Acids Res.* 2013;41(Database issue):D955-961. doi.org/10.1093/nar/gks1111
