# Supplementary material for: A novel adenoma–carcinoma sequence- and disulfidptosis-associated gene signature for prognostic prediction and mechanistic insights in colorectal cancer
Source: Genes Dis. 2026 Jun 3;14(2):102269. doi: 10.1016/j.gendis.2026.102269 (PMC13634562; doi:10.1016/j.gendis.2026.102269)
Supplement: Supplementary file 3 [file mmc3.docx]

**Contents**

**Table S1. Clinicopathological characteristics of patients included in the CRC cohorts.**	**2**

**Table S2. Stepwise regression (backward AIC) selection and multivariable Cox regression results for ACS-DRGs in CRC.**	**3**

**Table S3. Multivariate Cox regression analysis of six prognostic ACS-DRGs in CRC.**	**4**

**Table S4. Predicted chemotherapy sensitivity to 198 drugs in high- and low-risk patient groups using the “calcPhenotype” algorithm.**	**5**

**Table S1.** **Clinicopathological characteristics of patients included in the CRC cohorts.**

| Features |  | TCGA COADREAD | GSE39582 | GSE41258 | GSE17538 |
| --- | --- | --- | --- | --- | --- |
| Sample Size |  | N = 582 | N = 579 | N = 185 | N = 232 |
| Status | Live | 463 | 385 | 73 | 73 |
|  | Dead | 119 | 194 | 102 | 49 |
| Age | ≥65 | 341 | 363 | 102 | 122 |
|  | <65 | 241 | 216 | 73 | 110 |
| Gender | Male | 317 | 260 | 98 | - |
|  | Female | 265 | 319 | 87 | - |
| Tumor Stage | Stage I | 106 | 37 | 28 | 28 |
|  | Stage II | 216 | 269 | 50 | 72 |
|  | Stage III | 175 | 209 | 49 | 76 |
|  | Stage IV | 84 | 30 | 58 | 56 |
|  | Unknown | 1 | 4 | 0 | 0 |
| T Stage | T1 | 19 | 12 | 4 | - |
|  | T2 | 103 | 48 | 34 | - |
|  | T3 | 396 | 376 | 134 | - |
|  | T4 | 62 | 119 | 13 | - |
|  | Tis | 1 | 3 | 0 | - |
|  | Unknown | 1 | 21 | 0 | - |
| N Stage | N0 | 329 | 311 | 95 | - |
|  | N1 | 144 | 136 | 47 | - |
|  | N2 | 106 | 100 | 43 | - |
|  | NX | 2 | 12 | 0 | - |
|  | Unknown | 1 | 20 | 0 | - |
| M Stage | M0 | 434 | 496 | 127 | - |
|  | M1 | 83 | 61 | 58 | - |
|  | MX | 58 | 2 | 0 | - |
|  | Unknown | 7 | 20 | 0 | - |

**Table S2.** **Stepwise regression (backward AIC) selection and multivariable Cox regression results for ACS-DRGs in CRC.**

| stepAIC (backward) | | | | | |
| --- | --- | --- | --- | --- | --- |
| Cox Model: SLC12A2 + CXCL2 + LGALS2 + TIMP1 + ETS2 + S100P + SELENOP | | | | | |
|  | | Df | | AIC | |
| SLC12A2 | | 1 | | 1305.2 | |
| <none> | |  | | 1306.7 | |
| CXCL2 | | 1 | | 1307.2 | |
| LGALS2 | | 1 | | 1307.3 | |
| ETS2 | | 1 | | 1307.6 | |
| SELENOP | | 1 | | 1308.4 | |
| TIMP1 | | 1 | | 1311.6 | |
| S100P | | 1 | | 1313.1 | |
| Cox Model: CXCL2 + LGALS2 + TIMP1 + ETS2 + S100P + SELENOP | | | | | |
|  | | Df | | AIC | |
| <none> | |  | | 1305.2 | |
| LGALS2 | | 1 | | 1305.7 | |
| CXCL2 | | 1 | | 1306.2 | |
| SELENOP | | 1 | | 1307.1 | |
| ETS2 | | 1 | | 1308.7 | |
| TIMP1 | | 1 | | 1310.2 | |
| S100P | | 1 | | 1311.5 | |
|  | coef | exp(coef) | se(coef) | z | p |
| CXCL2 | -0.11931 | 0.88753 | 0.06874 | -1.736 | 0.08263 |
| LGALS2 | -0.09461 | 0.90973 | 0.05954 | -1.589 | 0.11202 |
| TIMP1 | 0.31272 | 1.36714 | 0.11797 | 2.651 | 0.00803 |
| ETS2 | -0.29135 | 0.74725 | 0.12106 | -2.407 | 0.0161 |
| S100P | -0.19216 | 0.82517 | 0.06304 | -3.049 | 0.0023 |
| SELENOP | -0.135 | 0.87371 | 0.06808 | -1.983 | 0.04738 |

***** Stepwise regression was performed using the backward Akaike information criterion (AIC) method to identify prognostic ACS-DRGs. The final model retained six genes: CXCL2, LGALS2, TIMP1, ETS2, S100P, and SELENOP. Abbreviations: Df, degrees of freedom; coef, regression coefficient; exp(coef), hazard ratio (HR); se(coef), standard error of the coefficient; z, Wald statistic; p, p-value.

**Table S3. Multivariate Cox regression analysis of six prognostic ACS-DRGs in CRC.**

|  | coef | exp(coef) | se(coef) | z | p |
| --- | --- | --- | --- | --- | --- |
| CXCL2 | -0.15355 | 0.85766 | 0.08592 | -1.787 | 0.0739 |
| LGALS2 | -0.06623 | 0.93592 | 0.07227 | -0.916 | 0.3595 |
| TIMP1 | 0.30392 | 1.35516 | 0.14101 | 2.155 | 0.0311 |
| ETS2 | -0.26654 | 0.76603 | 0.15156 | -1.759 | 0.0786 |
| S100P | -0.16196 | 0.85047 | 0.0795 | -2.037 | 0.0416 |
| SELENOP | -0.11651 | 0.89002 | 0.08494 | -1.372 | 0.1701 |

* Values represent the regression coefficient (coef), hazard ratio (exp(coef)), standard error (se(coef)), Wald statistic (z), and p-value for each gene. Risk scores were calculated as: Risk score = CXCL2 × (−0.15355) + LGALS2 × (−0.06623) + TIMP1 × 0.30392 + ETS2 × (−0.26654) + S100P × (−0.16196) + SELENOP × (−0.11651).

**Table S4. Predicted chemotherapy sensitivity to 198 drugs in high- and low-risk patient groups using the “calcPhenotype” algorithm.**

| Drug | P-value | Fold Change | Change | Mean sensitivity of high-risk group | Mean sensitivity of low-risk group |
| --- | --- | --- | --- | --- | --- |
| Cediranib_1922 | 0.044524841 | 1.085803094 | up | 9.845363527 | 9.067356304 |
| AMG-319_2045 | 0.04312836 | 1.074680352 | up | 143.2920477 | 133.3345747 |
| Mitoxantrone_1810 | 0.042452733 | 0.859424021 | down | 2.38786039 | 2.778442691 |
| Pyridostatin_2044 | 0.041210329 | 1.071473027 | up | 31.73967729 | 29.62246971 |
| LJI308_2107 | 0.041064927 | 1.059512234 | up | 172.8930012 | 163.1816941 |
| I-BET-762_1624 | 0.039034612 | 1.073322853 | up | 30.86680247 | 28.75817131 |
| Fludarabine_1813 | 0.03470842 | 1.090168613 | up | 175.8244293 | 161.2818671 |
| Rapamycin_1084 | 0.033197123 | 1.10277915 | up | 0.137951812 | 0.125094686 |
| Buparlisib_1873 | 0.029992343 | 1.074712454 | up | 2.907185896 | 2.705082541 |
| ERK_6604_1714 | 0.028830088 | 0.917750981 | down | 32.58060661 | 35.50048682 |
| Palbociclib_1054 | 0.02873332 | 1.118662989 | up | 47.39352419 | 42.36622168 |
| Venetoclax_1909 | 0.025661734 | 1.077890543 | up | 9.594186074 | 8.900890851 |
| SCH772984_1564 | 0.022434069 | 1.134182665 | up | 17.35399234 | 15.30087955 |
| PD0325901_1060 | 0.01785401 | 1.115181346 | up | 1.984317233 | 1.779367311 |
| Dasatinib_1079 | 0.01742436 | 0.874549773 | down | 5.917980315 | 6.766887941 |
| Topotecan_1808 | 0.015820144 | 0.859197784 | down | 1.276279052 | 1.485431033 |
| Dactinomycin_1811 | 0.01535889 | 1.13472397 | up | 0.10817524 | 0.095331766 |
| Ruxolitinib_1507 | 0.014960221 | 1.088838079 | up | 141.5702738 | 130.0195837 |
| SB216763_1025 | 0.014647277 | 1.058555513 | up | 205.6153932 | 194.2414834 |
| Docetaxel_1819 | 0.013787799 | 1.400953301 | up | 0.228879544 | 0.163374142 |
| ZM447439_1050 | 0.013419862 | 1.06787391 | up | 20.19012259 | 18.90684135 |
| KU-55933_1030 | 0.007639462 | 1.089755546 | up | 86.95163362 | 79.79003545 |
| AZD5363_1916 | 0.007384872 | 1.231932166 | up | 25.44912508 | 20.65789479 |
| Leflunomide_1578 | 0.006276049 | 1.066675709 | up | 157.8107372 | 147.946312 |
| Vinorelbine_2048 | 0.005719211 | 1.403626757 | up | 0.084294885 | 0.060055057 |
| Axitinib_1021 | 0.005655776 | 1.070419451 | up | 22.98849931 | 21.47615992 |
| PRT062607_1631 | 0.005476142 | 0.910349079 | down | 26.66786838 | 29.29411254 |
| Entinostat_1593 | 0.005443972 | 1.127871213 | up | 10.46369848 | 9.27738767 |
| Uprosertib_2106 | 0.005228896 | 1.433477233 | up | 24.3141736 | 16.96167406 |
| AZD4547_1786 | 0.005015493 | 1.132701172 | up | 21.57864988 | 19.05061142 |
| MG-132_1862 | 0.004609381 | 1.05897595 | up | 0.209067155 | 0.197423893 |
| Pevonedistat_1529 | 0.004067245 | 1.290725275 | up | 3.316750819 | 2.569679918 |
| JAK_8517_1739 | 0.003854066 | 0.832264909 | down | 23.4279643 | 28.14964809 |
| IRAK4_4710_1716 | 0.003846369 | 1.068065627 | up | 148.5602853 | 139.0928436 |
| BDP-00009066_1866 | 0.003436664 | 1.109064572 | up | 12.08464644 | 10.89625144 |
| Vinblastine_1004 | 0.003391732 | 1.65067337 | up | 0.052770851 | 0.031969287 |
| Podophyllotoxin bromide_1825 | 0.002719614 | 1.181078158 | up | 0.666242822 | 0.56409715 |
| PAK_5339_1730 | 0.002690912 | 1.073992119 | up | 11.67282592 | 10.86863275 |
| Vincristine_1818 | 0.002386898 | 1.564679136 | up | 0.40150389 | 0.256604617 |
| MIM1_1996 | 0.002380309 | 1.120741959 | up | 56.03425777 | 49.9974658 |
| MK-2206_1053 | 0.002111589 | 1.205034542 | up | 24.90802127 | 20.66996456 |
| Vorinostat_1012 | 0.002100491 | 1.098373433 | up | 4.61933047 | 4.205610161 |
| BMS-754807_2171 | 0.002048619 | 0.803077257 | down | 1.836481348 | 2.286805325 |
| AZ960_1250 | 0.001988597 | 0.843538066 | down | 8.768046343 | 10.39436949 |
| GSK269962A_1192 | 0.001683665 | 1.083574967 | up | 18.90673958 | 17.44848316 |
| Wnt-C59_1622 | 0.001499575 | 1.085932357 | up | 74.55221886 | 68.65272809 |
| AZD2014_1441 | 0.001291041 | 0.881419523 | down | 8.250156838 | 9.36007953 |
| SB505124_1194 | 0.001255317 | 1.079182987 | up | 10.34631378 | 9.5871728 |
| MK-8776_2046 | 0.001226295 | 1.250712017 | up | 31.04604387 | 24.82269575 |
| 5-Fluorouracil_1073 | 0.001196591 | 1.325699788 | up | 178.5106861 | 134.6539297 |
| Paclitaxel_1080 | 0.001195514 | 1.485490609 | up | 0.11348941 | 0.076398605 |
| Docetaxel_1007 | 0.00111329 | 1.27521481 | up | 0.015771536 | 0.012367748 |
| AZD1208_1449 | 0.000895538 | 1.119590732 | up | 219.3702032 | 195.9378521 |
| VX-11e_2096 | 0.000832153 | 1.166421472 | up | 20.65106861 | 17.70463688 |
| Dinaciclib_1180 | 0.00080268 | 1.181124106 | up | 0.075388325 | 0.063827606 |
| PF-4708671_1129 | 0.00078197 | 1.086408406 | up | 51.13989412 | 47.07243966 |
| Lapatinib_1558 | 0.000780352 | 1.184462596 | up | 24.73033885 | 20.87895298 |
| AZD8055_1059 | 0.000774094 | 0.956402588 | down | 0.811268372 | 0.848249871 |
| Oxaliplatin_1089 | 0.00068173 | 1.426235407 | up | 63.32145815 | 44.39762036 |
| IWP-2_1576 | 0.000673348 | 1.088269047 | up | 17.41638468 | 16.00374901 |
| ERK_2440_1713 | 0.000668467 | 0.848044788 | down | 15.30354098 | 18.04567542 |
| Ibrutinib_1799 | 0.000666635 | 1.151209933 | up | 106.1885656 | 92.24083513 |
| Daporinad_1248 | 0.000541121 | 1.506717044 | up | 0.019804348 | 0.013144039 |
| PFI3_1620 | 0.000457961 | 1.086293897 | up | 204.4198667 | 188.1809953 |
| Sapitinib_1549 | 0.000418417 | 1.125518763 | up | 58.78206859 | 52.22664476 |
| WZ4003_1614 | 0.000410267 | 0.878908279 | down | 42.8034168 | 48.70066403 |
| CDK9_5576_1708 | 0.000401258 | 1.22146046 | up | 0.869700263 | 0.712016714 |
| BI-2536_1086 | 0.000374156 | 1.232753181 | up | 1.686778398 | 1.368301801 |
| Osimertinib_1919 | 0.000247114 | 1.183014515 | up | 6.803525581 | 5.751007696 |
| VE-822_1613 | 0.000238442 | 1.197759495 | up | 34.79524403 | 29.05027611 |
| Tozasertib_1096 | 0.000225631 | 1.16108137 | up | 21.71334002 | 18.70096325 |
| AZD6738_1917 | 0.000163681 | 1.376961022 | up | 11.22252215 | 8.150210478 |
| IGF1R_3801_1738 | 0.000159313 | 0.809935224 | down | 5.680005093 | 7.012912789 |
| PD173074_1049 | 0.000103551 | 1.230627478 | up | 73.24696501 | 59.52001424 |
| Cytarabine_1006 | 8.64E-05 | 1.385561333 | up | 8.671981168 | 6.258821578 |
| YK-4-279_1239 | 7.77E-05 | 1.653182792 | up | 18.28497766 | 11.06046938 |
| LY2109761_1852 | 7.53E-05 | 1.143733935 | up | 197.2676674 | 172.4768859 |
| Nelarabine_1814 | 6.29E-05 | 1.138161728 | up | 466.1652468 | 409.5773346 |
| XAV939_1268 | 5.03E-05 | 0.90506115 | down | 80.51143652 | 88.9569026 |
| Temozolomide_1375 | 4.36E-05 | 1.183311026 | up | 477.7259116 | 403.7196485 |
| AZD5438_1401 | 4.36E-05 | 1.297838925 | up | 12.00466412 | 9.24973345 |
| MN-64_1854 | 2.61E-05 | 1.149703995 | up | 127.4475978 | 110.8525311 |
| AT13148_2170 | 2.41E-05 | 1.294652338 | up | 50.89849476 | 39.31441149 |
| EPZ004777_1237 | 2.01E-05 | 1.178907989 | up | 199.3341165 | 169.0836929 |
| Wee1 Inhibitor_1046 | 1.21E-05 | 1.332348465 | up | 10.26031067 | 7.7009213 |
| AGI-6780_1634 | 9.18E-06 | 1.130955725 | up | 68.54062022 | 60.60415866 |
| MK-1775_1179 | 9.14E-06 | 1.354386516 | up | 2.42167033 | 1.788020112 |
| AZD6482_2169 | 8.15E-06 | 1.120759142 | up | 26.67895536 | 23.80436113 |
| I-BRD9_1928 | 5.23E-06 | 1.214195075 | up | 95.87423808 | 78.96114885 |
| VSP34_8731_1734 | 4.45E-06 | 1.167440211 | up | 12.31744147 | 10.55081138 |
| Ipatasertib_1924 | 4.36E-06 | 1.311622654 | up | 44.18389028 | 33.68643424 |
| Ulixertinib_2047 | 4.04E-06 | 1.221653943 | up | 11.09070451 | 9.078433847 |
| GSK343_1627 | 3.77E-06 | 1.10756271 | up | 17.91290788 | 16.17326741 |
| Bortezomib_1191 | 2.86E-06 | 1.154461963 | up | 0.008654155 | 0.007496267 |
| Picolinici-acid_1635 | 2.43E-06 | 1.105869281 | up | 182.227917 | 164.7825111 |
| Ulixertinib_1908 | 1.94E-06 | 1.206870812 | up | 19.33602926 | 16.02162309 |
| Fulvestrant_1816 | 1.31E-06 | 1.166136505 | up | 106.7732297 | 91.56151895 |
| GSK2578215A_1927 | 8.72E-07 | 1.150075104 | up | 150.9645138 | 131.2649177 |
| BIBR-1532_2043 | 8.33E-07 | 1.154016274 | up | 159.0911403 | 137.8586628 |
| RO-3306_1052 | 6.54E-07 | 1.218585452 | up | 17.91792625 | 14.70387343 |
| Acetalax_1804 | 5.40E-07 | 1.283142604 | up | 172.5992972 | 134.5129502 |
| Afatinib_1032 | 5.38E-07 | 1.210968342 | up | 7.300236287 | 6.028428685 |
| Sorafenib_1085 | 3.25E-07 | 1.249670417 | up | 17.14350415 | 13.71842041 |
| AZD1332_1463 | 3.18E-07 | 0.816247685 | down | 47.79887618 | 58.55927931 |
| BMS-345541_1249 | 1.90E-07 | 1.313062694 | up | 37.82377337 | 28.80576346 |
| Zoledronate_1802 | 1.85E-07 | 1.167097359 | up | 48.82112831 | 41.83123879 |
| Afuresertib_1912 | 1.11E-07 | 1.380022629 | up | 16.77800809 | 12.15777752 |
| KRAS (G12C) Inhibitor-12_1855 | 7.93E-08 | 1.385799854 | up | 112.807971 | 81.40278745 |
| EPZ5676_1563 | 5.97E-08 | 1.234723381 | up | 303.7365119 | 245.9955943 |
| Tamoxifen_1199 | 5.74E-08 | 1.192361702 | up | 40.90418368 | 34.30518074 |
| AGI-5198_1913 | 5.61E-08 | 1.151003959 | up | 116.0849495 | 100.8553868 |
| IAP_5620_1428 | 5.47E-08 | 1.27309705 | up | 210.8250961 | 165.6001765 |
| VE821_2111 | 2.73E-08 | 1.33899462 | up | 79.48018799 | 59.35810853 |
| Carmustine_1807 | 9.11E-09 | 1.203716065 | up | 518.997946 | 431.1630967 |
| LGK974_1598 | 6.70E-09 | 1.245987914 | up | 67.75938414 | 54.3820557 |
| Nilotinib_1013 | 6.53E-09 | 1.282240122 | up | 45.27833764 | 35.31190209 |
| MIRA-1_1931 | 5.11E-09 | 1.263341178 | up | 273.8602109 | 216.7745464 |
| Crizotinib_1083 | 2.42E-09 | 1.345966661 | up | 31.66112852 | 23.52296638 |
| Fulvestrant_1200 | 2.14E-09 | 1.192848032 | up | 21.04119308 | 17.63945827 |
| AZD5991_1720 | 8.96E-10 | 1.880084652 | up | 135.0574566 | 71.83583806 |
| P22077_1933 | 7.48E-10 | 1.3921139 | up | 117.5568799 | 84.4448719 |
| LCL161_1557 | 6.93E-10 | 1.222380279 | up | 162.8595861 | 133.2315229 |
| GSK1904529A_1093 | 5.59E-10 | 1.231955993 | up | 88.32806683 | 71.69742046 |
| Savolitinib_1936 | 4.22E-10 | 1.276416719 | up | 16.85649003 | 13.20610251 |
| Cyclophosphamide_1512 | 4.22E-10 | 1.218434678 | up | 202.1936887 | 165.9454482 |
| Sinularin_1838 | 3.99E-10 | 1.222316252 | up | 41.76317547 | 34.16724222 |
| Gefitinib_1010 | 7.94E-11 | 1.211817793 | up | 29.27128345 | 24.15485531 |
| ML323_1629 | 5.64E-11 | 1.250232256 | up | 104.8285469 | 83.84725824 |
| GSK591_2110 | 3.30E-11 | 1.253757167 | up | 114.4928073 | 91.31976295 |
| GDC0810_1925 | 1.43E-11 | 1.268320762 | up | 163.4265379 | 128.8526868 |
| OF-1_1853 | 1.54E-13 | 1.300503803 | up | 73.61626749 | 56.60596097 |
| AZD3759_1915 | 1.43E-13 | 1.222674007 | up | 16.5003698 | 13.49531413 |
| Gallibiscoquinazole_1830 | 5.54E-14 | 1.274519592 | up | 16.02859233 | 12.57618355 |
| Erlotinib_1168 | 5.20E-14 | 1.282568388 | up | 16.27825182 | 12.69191722 |
| TAF1_5496_1732 | 3.72E-15 | 1.416494948 | up | 59.412756 | 41.94350009 |
| Dihydrorotenone_1827 | 1.37E-17 | 1.329366754 | up | 2.94837725 | 2.217880988 |

* Drugs with a positive fold change (“up”) indicate higher predicted sensitivity in the high-risk group, whereas a negative fold change (“down”) indicates higher predicted sensitivity in the low-risk group. Mean sensitivity values are shown for both high- and low-risk groups.
